# The prevalence of high blood pressure and its determinants among Tunisian adolescents

**DOI:** 10.1038/s41371-022-00677-x

**Published:** 2022-04-08

**Authors:** Sarra Soua, Rim Ghammam, Jihene Maatoug, Nawel Zammit, Sihem Ben Fredj, Fernando Martinez, Hassen Ghannem

**Affiliations:** 1 Université de Sousse, Faculté de Médecine de Sousse, 4000 Sousse, Tunisia; 2 Hôpital Farhat Hached, Service d’Epidémiologie, «LR19SP03», 4000 Sousse, Tunisia; 3 Cardiometabolic and renal research group, Research Foundation of the Clinical Hospital of Valencia, Valencia, Spain; 4 University of Valencia, Valencia, Spain

**Keywords:** Hypertension, Diagnosis, Lifestyle modification, Preventive medicine

## Abstract

Hypertension can originate in childhood and remain undetected unless special screening is performed. The burden of hypertension in adolescents in Tunisia is unknown. The aim of this study was to investigate the prevalence of blood pressure (BP) within the hypertension range and its association with other risk factors among Tunisian adolescents. A cross-sectional study that included 1385 secondary school students in Sousse, Tunisia, was performed during 2017–2018. A two-stage cluster sampling strategy was used to obtain a representative sample of the study population. BP within the hypertension range (HBP)was classified following the European guideline recommendations for measuring BP in children and adolescents. Anthropometric indices were measured using a standard protocol. A structured questionnaire collected information about sociodemographic characteristics, lifestyle, mental health status, and addictions. Adjusted logistic regression models were used to assess hypertension-related risk factors. Our study included 39.5% boys and 60.5% girls. The mean age of our population was 17 ± 1.5 years. The prevalence of HBP was 15.4% (13.1–18.0%), and it was significantly higher in boys (22.8%) than in girls (10.6%, *p* value < 0.001). In the multivariate logistic regression model, overweight [OR = 1.72(1.18–2.51)] and obesity [OR = 3.73(2.55–5.41)] were independent risk factors for HBPrange, (*p* value < 0.001), whereas female sex [OR = 0.41(0.29–0.56), *p* value < 0.001] and depression [OR = 0.67(0.51–0.88), *p* value = 0.008] were independent protective factors. Among Tunisian secondary school adolescents, the prevalence of HBP was high and associated with excess body weight. A comprehensive strategy for the prevention of hypertension and its risk factors among youth is urgently needed.

## Introduction

Hypertension has become a global pandemic. Hypertension affects approximately 1 billion adults and is associated with more than 9 million deaths annually. Although hypertension was previously known to be a disease for adults 40 years old or older, it is not uncommon in children and adolescents [1]. Hypertension is a significant concern because of its rising prevalence and because evidence suggests that hypertension can begin in childhood [1].

Hypertension has a potential impact on health, exposing adolescents to cardiovascular and kidney damage, arterial wall dysfunction, and cognitive changes [1]. Hypertension is also associated with an increased risk of premature mortality in adulthood [2]. Numerous studies suggest that elevated BP in childhood increases the future risk of left ventricular hypertrophy [1], carotid intima-media thickness (IMT), and atherosclerosis [3]. Most importantly, hypertension is preventable if relatively simple measures such as maintaining a healthy lifestyle and avoiding certain risk factors are followed. The main modifiable risk factors of hypertension are overweight and obesity, physical inactivity, smoking, unhealthy diet, and high salt and alcohol intake. Studies also show that psychosocial factors, such as environmental stress, play a role in developing high blood pressure (HBP) and are subject to culturally sensitive interventions for hypertension prevention [4]. These facts outline the need to start hypertension screening and study its associated factors beginning at early ages to prevent later complications.

Tunisia, a North African low-middle-income country (LMIC), is experiencing a demographic and epidemiological transition characterized by a decline in birth and death rates, an increase in life expectancy, the regression of infectious diseases, and the emergence of noncommunicable diseases [5]. This change has affected lifestyle habits, especially in children and young people, who have become more inactive and obese

Tunisia adolescent population is not different to other North African LMICs with a mixed ethnicity as a result of different genetic backgrounds, including Arab, Berber, Turkish, and Andalusian. Mediterranean diet rich in fibre, fruits, and vegetables, is being replaced with Western diet characterised by an increased consumption of fast food, and technological hobbies have replaced outdoor activities.

In Tunisian adolescents, the prevalence of HBP and its related factors, including psychosomatic dimensions, is currently unknown. Screening for hypertension in this healthy population may help with adequate prevention and better health resource allocation and policy-making.

Therefore, this study aimed to determine the prevalence of BP within the hypertension range (HBP) and its determinants among upper secondary school adolescents in Sousse, Tunisia.

## Methodology

### Study design

This cross-sectional study was conducted in the urban area of the region of Sousse in Tunisia during the 2017-2018 school year. The governorate of Sousse is divided into sixteen geographic areas. Three of these geographic areas represent the city/town of Sousse: Sousse-Medina, Sousse-Jawhra, and Sousse-Riyadh.

### Study population and sampling design

The study population included upper secondary school adolescents who were between 14 and 19 years old in Tunisia. The sample size was calculated using the free software Epi Info^TM^ version6. The sample size was based on the estimated prevalence of smoking status, previously reported as 26% in this population, as a more prevalent risk factor than the estimated prevalence of hypertension in adolescents in LMICs [6]. The calculation considered an α error of 5%, a precision of 4.5%, and a cluster effect of 2. Thus, for this study, the estimated sample size was 1095 participants. The required sample size, assuming a 20% nonresponse rate, was 1317 adolescents.

To obtain a representative sample of the study population, a stratified two-stage sampling design was used.

Sousse City is divided into three geographic areas: Sousse-Medina, Sousse-Jawhara, and Sousse-Riyadh. In these geographic areas, 10 out of 12 secondary schools were eligible for the study.

One or two secondary schools within each geographic area were randomly selected in the first stage depending on the student population density (number of students >500). Four out of a total of ten eligible secondary schools were randomly selected.

In the second stage, classes in the selected secondary schools were stratified by grades (1st, 2nd, 3rd, or 4th year). Then, from each grade, a number of classes were randomly selected. Finally, 59 classes were selected across schools to obtain the desired sample size. Only students who agreed to participate were finally included.

### Data collection

One week before the beginning of data collection, parents were informed and asked to give their written consent. Data collection took place in classrooms during regular class times. First, the study purposes were explained to students by trained research staff, and students were assured about data confidentiality and anonymity. Then, a validated questionnaire in Arabic was distributed and completed by students in the presence of investigators. The data collection period lasted five months, from January to May 2018.

### Measurements

Sociodemographic characteristics, lifestyle behaviours, and addictions were evaluated using validated self-administered questionnaires originally in Arabic or translated (Supplementary material). The collected information included age, sex, educational level, parents’ occupation, parents’ education level, student’s weekly pocket money, dietary habits, physical activity (PA), sedentary behavior, smoking, drinking, illicit substance use, videogame addiction (VGA), and Facebook addiction (FA). Mental health, including self-esteem, depression, anxiety, and alexithymia, was also evaluated through validated questionnaires (Supplementary material).

For students, regular PA was defined as moderate PA through exercise for at least 60 min per day for at least 5 days per week. Sedentarism was defined by a set of behaviours during which the sitting or lying position was dominant and the energy expenditure was very low or even absent. The time spent on the internet and in front of a screen (television, video games, and computers) was used to indicate physical inactivity. We considered sedentary students to be those who spent at least 2 h per day on the internet.

The evaluation of dietary habits was based on an estimate of the adequate daily proportion of fruits and vegetables. According to the WHO report, the intake of 5 servings of fruits and vegetables or a minimum of 400 g per day is recommended.

An irregular school career was defined as having to repeat one year or more at school.

Smokers were those who had smoked at least one cigarette during the last month. Combined smokers were those who had vaped or smoked cigarettes or water pipes at least once during the last month.

Illicit substance use was defined as the consumption of at least one psychoactive substance (cannabis, cocaine, fentanyl, ecstasy) at least once during the last month.

Alcohol consumption was defined as the consumption of alcohol at least once in life.

VGA and FA were assessed with a 21-item DSM-based questionnaire and a brief version of the BFAS, respectively (Supplementary material).

Self-esteem was evaluated with the RSE questionnaire, depression with the Arabic version of the BDI-II scale, alexithymia with the TAS-20 tool and anxiety with the SCARED-C instrument (Supplementary material).

### Anthropometric measurements

Weight was measured to the nearest 0.1 kg using a portable electronic scale without shoes and with light clothing. Height was also measured without shoes to the nearest 0.5 cm using a portable stadiometer fixed on the wall with the student’s back against the wall and looking forwards. Body mass index (BMI) was calculated as weight (kg)/height² (m²). Students were classified as obese or overweight based on the IOTF criteria for adolescents if their BMI was equal to or greater than the threshold values for age and sex proposed by T J Cole et al. [7]. This article aimed to develop an international definition of child overweight and obesity according to age and sex. The survey included 6 large national cross sectional samples. A total of 97 876 males and 94 851 females were examined to calculate their BMI. The proposed cut off points are more representative and allow better comparability of overweight prevalence across the world [7].

### Blood pressure

All the staff in charge of taking the blood pressure (BP) was properly trained in the BP measurement procedure before the initiation of the study. The students’ pulse regularity was evaluated before the measurements. BP was measured in a quiet room, after 15 min of rest, in the sitting position. Students were positioned with their backs and arms supported. The cuff was placed in the nondominant arm with the arm positioned at the heart level after selecting an adequate cuff size based on the students’ arm circumference. A validated electronic sphygmomanometer device (Omron M3 Intellisense HEM‑7051‑E, OMRON Healthcare Co., Ltd, Kyoto, Japan) was used [8]. Students were instructed not to smoke or have any stimulants during the previous hour. Due to logistical reasons and for screening purposes, BP was measured only once. If the first BP reading was within the high-normal or hypertension range, a second BP measurement was repeated after 10 min of rest. In this case, the lowest BP was used to classify the student. BP was classified based on the European Society of Hypertension (ESH) Guidelines on HBP in Children and Adolescents published in 2016 (ref. [9]). The definition of hypertension was also based on the 2016 ESH Guidelines [9]. These guidelines used the normative data on auscultatory clinic measurements provided by the US Task Force, providing BP percentiles for each sex, ages from 1 to 17 years, and for seven height percentile categories. For adolescents aged under 16, HBP was defined as systolic BP (SBP) and/or diastolic BP (DBP) persistently at no less than the 95th percentile for sex, age, and height. For adolescents aged 16 years old or above, the definition was based on the absolute cut-off used for adults.Children with HBP were referred to their paediatricians for reassessment.

### Statistical analysis

All the analyses were assessed considering the complex survey design characteristics with the sampling weights and after applying the finite population correction to obtain the most reliable estimators.

Quantitative variables are expressed as the population means and linearized standard errors. Qualitative variables are expressed as the population proportions and 95% confidence bounds for cell proportions. The interquartile range was used to exclude extreme outliers. Comparisons between independent groups were made by the chi-square test or ANOVA for categorical and continuous variables, respectively. The significance level was set at a *p* value < 0.05.

Pearson correlation was used to assess the relationship between BMI and BP deciles. Logistic regression models, adjusted by age, sex, and BMI categories, were used to determine the independent factors related to HBP. The results were expressed using the adjusted odds ratio (aOR) and its 95% confidence interval. Variables with a *p* value ≤ 0.2 in the univariate analysis were further considered in the model. The variance inflation factor (VIF) was used to assess collinearity among the study variables. The statistical analysis was carried out using the module Complex Samples with the programme SPSS v.25. for Windows (Statistical Package for the Social Sciences, International Business Machines, Inc., Armonk, New York, USA).

### Ethical considerations

The study protocol was approved by the Farhat Hached University Hospital ethical committee, Sousse, Tunisia. An official authorization was obtained from the Regional Directorate of education, the school administrators, and teachers. Written informed consent was obtained from parents before data collection for those aged less than 18 years old.

Investigators were instructed not to make any comments on the use of substances before the survey and not to look into students’ sheets while they were filling in the questionnaires. To further assure student data confidentiality, data were anonymized prior to the analysis.

## Results

The analysis included one thousand three hundred eighty-five students from four secondary schools, 1 from Sousse-Medina, 2 from Sousse-Jawhra, and 1 from Sousse-Riyadh (mean age 17.0 ± 0.3 years, 60.5% females, mean BMI 22.7 ± 0.2 kg/m^2^). The overall response rate was 86.68%, and all the included students were from the same ethnic group. Seven students were excluded as extreme outliers because of SBP higher than 182 or DBP higher than 120 mmHg.

### Blood pressure distribution

**Fig. 1:**
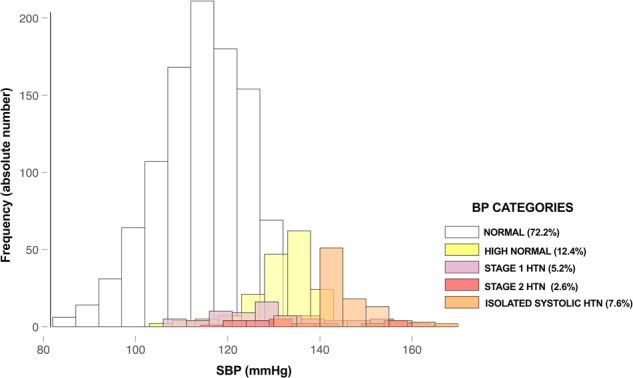
Overlapping histogram for BP categories. Note: Although not shown in the graph, the total proportions shown in the figure also include students with hypertensiondue to the high diastolic BP component.

After excluding outliers, BP followed a normal distribution (Fig. 1). The mean systolic and diastolic BP were 119.3 ± 0.6 mmHg and 70.2 ± 0.3 mmHg, respectively. There were significant BP differences between boys and girls (mean SBP was 124.4 ± 0.6 mmHg in males vs. 116.1 ± 0.4 mmHg in females, *p* value < 0.001; mean DBP was 69 ± 0.5 mmHg in males vs. 71 ± 0.40 mmHg in females; *p* value = 0.004).

The proportion of students with HBP was 15.4% (13.1–18.0%) and was significantly higher for boys than girls (22.8% vs. 10.6%, *p* value < 0.001). Additionally, 12.4% had BP values within the high-normal range. The largest percentage of students with BP fell within the isolated systolic hypertension (ISH) category (7.6%), followed by stage 1 (5.2%) and stage 2 hypertension(2.6%). HBP was due to high DBP only in 30% of the students. Although the average SBP was 1.7 mmHg lower in those younger than 16 years, the prevalence of HBP was significantly higher in this group than in the older group (23.4 vs. 13.8%; *p* value < 0.001). The main characteristics of the study population according to BP categories are shown in Table 1. Figure 1 shows the histograms of BP categories after the exclusion of outliers.

**Table 1:** Sociodemographic characteristics according to BP categories in secondary school adolescents in Sousse, Tunisia, 2017–2018.

	TOTAL *n* (%)	HTN			BP CATEGORIES n(%)					
		No *N* (%)	Yes *N* (%)	*p* value	Normal	High normal	Stage 1HTN	Stage 2 HTN	ISH	*p* value
**Age categories**										
<16	257(18.5)	198(16.9)	59(27.8)	0.001	172(17.2)	26(15.2)	22(30.6)	10(28.6)	27(25.7)	0.004
≥16	1130(81.5)	975(83.1)	153(72.2)		830(82.8)	145(84.8)	50(69.4)	25(71.4)	78(74.3)	
**Sex**										
Male	548(39.5)	423(36.1)	125(59.0)	<0.001	343(34.2)	80(46.8)	31(43.0)	17(48.6)	77(73.3)	<0.001
Female	839(60.5)	750(63.9)	87(41.0)		659(65.8)	91(53.2)	41(57.0)	18(51.4)	28(26.7)	
**Class**										
First year	366(26.4)	300(25.6)	65(30.7)	0.453	264(24.9)	36(21.1)	26(36.1)	10(28.6)	29(27.6)	0.088
Second year	302(21.8)	261(22.2)	41(19.3)		232(21.8)	29(17.0)	13(18.1)	7(20.0)	21(20.0)	
Third year	350(25.2)	298(25.4)	52(245)		295(27.8)	63(36.8)	15(20.8)	13(37.1)	24(22.9)	
Fourth year	369(26.6)	314(26.8)	54(25.5)		271(25.5)	43(25.1)	18(25.0)	5(14.3)	31(29.5)	
**Irregular school career**										
Yes	402(29.0)	354(30.2)	47(22.2)	0.053	306(30.5)	48(28.1)	18(25.0)	7(20.0)	22(20.9)	0.121
No	985(71.0)	819(69.8)	165(77.8)		696(69.5)	123(71.9)	54(75.0)	28(80.0)	83(79.1)	
**Educational level of mothers**										
Primary education	385(27.8)	339(29.1)	46(22.1)	0.018	287(28.9)	52(30.4)	16(22.9)	7(20.0)	23(22.3)	0.033
Secondary education	486(35.0)	414(35.5)	72(34.6)		364(36.6)	50(29.2)	25(35.7)	15(42.9)	32(31.1)	
Higher Education	504(36.3)	412(35.4)	90(43.3)		343(34.5)	69(40.3)	29(41.4)	13(37.1)	48(46.6)	
**Educational level of fathers**										
Primary education	325(23.4)	286(24.6)	39(18.7)	0.083	232(23.3)	54(32.0)	15(21.1)	7(20.0)	17(16.5)	0.019
Secondary education	476(34.3)	402(34.6)	73(34.9)		353(35.5)	49(29.0)	32(45.1)	11(31.4)	30(29.1)	
Higher Education	573(41.3)	475(40.8)	97(46.4)		409(41.2)	66(39.0)	24(33.8)	17(48.6)	56(54.4)	
**Mothers’ profession**										
Unemployed	698(50.3)	603(51.9)	95(45.7)	0.209	514(51.8)	89(52.3)	31(43.6)	20(57.1)	44(43.1)	0.178
Labourer	119(8.6)	99(8.5)	20(9.6)		91(9.2)	8(4.7)	7(9.9)	2(5.7)	11(10.8)	
Private sector	105(7.6)	91(7.8)	14(6.7)		80(8.1)	11(6.5)	8(11.3)	2(5.7)	4(3.9)	
Employee	326(23.5)	266(22.9)	59(28.4)		217(21.9)	49(28.8)	19(26.8)	7(20.0)	33(32.3)	
Senior staff	124(8.9)	103(8.9)	20(9.6)		90(9.1)	13(7.7)	6(8.4)	4(11.4)	10(9.8)	
**Fathers’ profession**										
Unemployed	81(5.8)	72(6.6)	9(11.1)	0.742	61(3.6)	11(3.8)	4(3.1)	2(3.7)	3(1.8)	0.246
Labourer	207(14.9)	178(16.3)	29(14.1)		156(9.1)	22(7.6)	10(7.9)	5(9.2)	14(8.3)	
Private sector	375(27.0)	318(29.1)	56(14.9)		266(15.6)	52(18.1)	26(20.5)	9(16.7)	21(12.4)	
Employee	396(28.6)	333(30.5)	62(15.8)		286(16.8)	47(16.3)	20(15.7)	9(16.7)	33(19.5)	
Senior staff	229(16.5)	191(17.5)	38(16.6)		936(54.9)	156(54.2)	67(52.8)	29(53.7)	98(58.0)	
**Pocket money per week**										
<10 DT	510(40.6)	437(41.0)	72(38.1)	0.277	377(41.5)	60(38.5)	29(46.0)	11(37.9)	32(33.0)	0.284
≥10 DT	746(59.4)	628(59.0)	117(61.9)		532(58.5)	96(61.5)	34(54.0)	18(62.1)	65(67.0)	

*Significant differences among groups are highlighted in bold type.

### Associated factors for hypertension

BMI and weight status

**Fig. 2:**
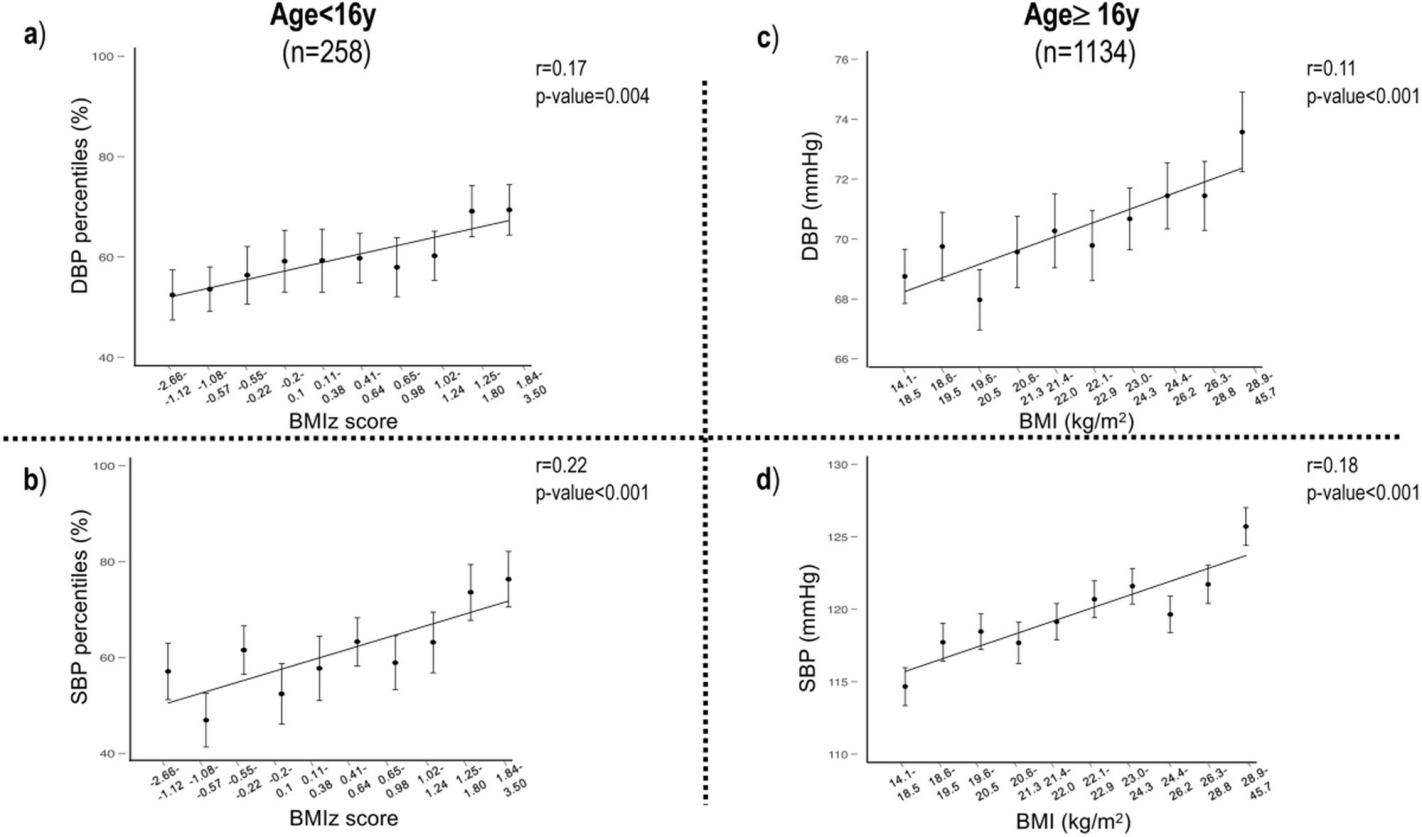
Estimated regression line. **a** Estimated regression line between deciles of the DBP percentile and BMIz score in students younger than 16 y. The mean BMIz score for each decile of the DBP percentile ±standard error is also shown. **b** Estimated regression line between deciles of the SBP percentile and BMIz score in students younger than 16y. The mean BMIz score for each decile of the SBP percentile ±standard error is also shown. **c** Estimated regression line between deciles of DBP and BMI in students 16 y of age or older. The mean BMI for each decile of DBP ± standard error is also shown. **d** Estimated regression line between deciles of SBP and BMI in students 16 y of age or older. The mean BMI for each decile of SBP±standard error is also shown.

There was a highly significant correlation between BP percentiles and BMIz score and between BP and BMI for those younger or older than 16 years (*r* = 0.21 and 0.25 for percentiles of diastolic and systolic BP with BMIz score; *r* = 0.14 and 0.20 for DBP and SBP with BMI, *p* value = 0.001). There was also a significant trend for higher BMI across BP levels, *p* value < 0.001. The estimated regression line between BP percentile/BP and BMIz score/BMI with the average BMIz score/BMI for ascending categories of BP percentile/BP is shown in Fig. 2.The prevalence of HBP in overweight-obese students was significantly higher than that in normal-weight students (22% vs. 12.9%, *p* value = 0.002, Table 1).

### Sociodemographic factors

The only sociodemographic factor associated with HBP was mothers’ educational level (*p* value = 0.018) (Table 1). In contrast, both mothers’ and fathers’ educational levels were associated with BP levels. Other factors, such as irregular school careers, parents’ occupation, and students’ class level, did not show differences across BP categories.

### Lifestyle habits and addictive behaviours

There were no significant differences across BP categories for lifestyle habits, including regular PA, time spent on the internet, fruit and vegetable intake (FVI), and fast-food consumption (Table 2).

**Table 2:** Lifestyle factors and addictive and mental behaviours according to BP categories among secondary school adolescents in Sousse, Tunisia, 2017–2018.

		HTN n(%)			BP CATEGORIES n(%)					
		No	Yes	*p* value	Normal	High- normal	Stage 1HTN	Stage 2 HTN	ISH	*p* value
**Weight status**	Normal	879(87.1)	129(12.9)	**0.002**	766(75.9)	113(11.3)	40(4.0)	21(2.1)	68(6.8)	**0.002**
	Overweight-obesity	294(78.0)	83(22.0)		236(62.6)	58(15.4)	32(8.4)	14(3.7)	37(9.9)	
**Physical activity**	Yes	481(84.6)	87(15.4)	0.952	401(70.5)	80(14.1)	32(5.6)	12(2.1)	43(7.6)	0.427
	No	671(84.8)	120(15.2)		582(73.5)	89(11.3)	39(4.9)	22(2.8)	59(7.5)	
**Time spent on the internet per day (hours)**	<=2	281(84.9)	50(15.1)	0.849	223(67.4)	58(17.5)	16(4.8)	8(2.4)	26(7.9)	0.096
	>2	811(84.4)	149(15.6)		708(73.6)	103(10.8)	49(5.1)	25(2.6)	75(7.9)	
**Consumption of 5 vegetables and fruits/day**	Yes	383(84.5)	70(15.5)	0.868	324(71.4)	59(13.1)	15(3.3)	11(2.4)	44(9.8)	0.063
	No	772(84.8)	138(15.2)		663(72.8)	109(12.0)	55(6.0)	23(2.5)	60(6.6)	
**Fast-food consumption**	≤3 days/week	854(83.4)	170(16.6)	0.084	724(70.6)	130(12.7)	56(5.5)	26(2.5)	88(8.7)	0.151
	>3 days/week	297(88.1)	40(11.9)		260(77.0)	37(11.1)	15(4.5)	8(2.4)	17(77.0)	
**Smoking**	Yes	159(83.2)	32(16.8)	0.532	133(69.5)	26(13.7)	9(4.7)	2(1.1)	21(11.1)	0.207
	No	978(84.8)	175(15.2)		838(72.6)	140(12.2)	59(5.1)	33(2.9)	83(7.2)	
**Electronic cigarettes /vape**	Yes	145(84.8)	26(15.2)	0.975	118(68.9)	27(15.8)	9(5.3)	2(1.2)	15(8.8)	0.445
	No	986(84.8)	176(15.2)		848(72.9)	138(11.9)	58(5.0)	33(2.8)	85(7.4)	
**Water pipe**	Yes	119(80.9)	28(19.1)	0.184	102(69.3)	17(11.6)	5(3.4)	1(0.7)	22(15.0)	**0.011**
	No	1019(84.9)	180(15.1)		871(72.6)	148(12.4)	65(5.4)	34(2.8)	81(6.8)	
**Combined smoking**	Yes	880(84.2)	157(15.8)	0.778	193(70.6)	37(13.6)	12(4.4)	2(0.7)	29(10.6)	0.121
	No	230(84.8)	43(15.2)		753(72.6)	127(12.3)	53(5.1)	33(3.2)	71(6.9)	
**Alcohol**	Yes	114(81.8)	25(18.2)	0.591	88(63.1)	26(18.7)	6(4.4)	3(2.2)	16(11.6)	0.148
	No	1048(84.8)	187(15.2)		904(73.1)	144(11.7)	66(5.3)	32(2.6)	89(7.3)	
**Illicit substance use**	Yes	82(84.3)	15(15.7)	0.888	69(70.9)	13(13.5)	5(5.2)	2(2.1)	8(8.3)	0.930
	No	1086(84.7)	196(15.3)		928(72.3)	158(12.4)	67(5.2)	33(2.6)	96(7.5)	
**Facebook addiction**	Yes	435(87.5)	62(12.5)	0.046	379(76.2)	56(11.2)	25(5.0)	7(1.4)	30(6.1)	**0.049**
	No	738(83.1)	150(16.9)		623(70.1)	115(13.0)	47(5.3)	28(3.1)	75(8.5)	
**Videogame addiction**	Yes	397(81.2)	91(18.8)	0.011	342(70.0)	55(11.3)	31(6.4)	9(1.9)	51(10.5)	**0.028**
	No	776(86.5)	1221(13.5)		660(73.5)	116(13.0)	41(4.5)	26(2.9)	54(6.1)	
**Depression**	Severe or moderate	645(88.5)	144(11.5)	0.002	455(76.3)	73(12.3)	28(4.7)	10(1.7)	30(5.1)	**0.042**
	Mild or absent	528(81.7)	68(18.3)		547(69.2)	98(12.5)	44(5.6)	25(3.2)	75(9.6)	
**Anxiety**	Yes	586(87.4)	84(12.6)	0.024	513(76.5)	73(11.0)	35(5.2)	12(1.8)	37(5.6)	0.056
	Possible	227(84.9)	40(15.1)		189(70.6)	38(14.3)	11(4.1)	7(2.6)	22(8.3)	
	Absent	360(80.4)	88(19.6)		300(67.0)	60(13.3)	26(5.8)	16(3.5)	46(10.3)	
**Alexithymia**	Yes	561(85.3)	96(14.7)	0.156	493(75.0)	68(10.3)	37(5.7)	17(2.6)	42(6.4)	**0.016**
	Possible	294(86.4)	46(13.6)		253(74.3)	41(12.1)	12(3.5)	6(1.8)	28(8.3)	
	None	318(82.0)	70(18.0)		256(65.9)	62(16.0)	23(5.9)	12(3.1)	35(9.1)	
**Self esteem**	High	263(84.3)	49(15.7)	0.821	215(68.8)	48(15.4)	14(4.4)	5(1.6)	30(9.7)	0.267
	Moderate/median	488(85.2)	85(14.8)		419(73.1)	69(12.1)	26(4.5)	17(3.0)	42(7.4)	
	Low	422(84.3)	78(15.7)		368(73.5)	54(10.8)	32(6.5)	13(2.6)	33(6.6)	

*Significant differences among groups are highlighted in bold type.

The prevalence of HBP in adolescents with a VGA was higher than that in those without a VGA (18.8% vs. 13.5%, *p* = 0.011); the opposite was true for Facebook addiction (12.5% vs. 16.9%, *p* = 0.046). Other addictions, including smoking, alcohol intake, and illicit substance use, were notably associated with HBP.

### Mental health

Regarding mental health, the prevalence of HBP range in euthymic students was higher than that in those with moderate or severe depression (18.3% vs. 11.5%, *p* value = 0.024). Similar results were found for anxiety, with a higher prevalence of HBP in those without anxiety than in those with anxiety. For other mental traits, such as self-esteem and alexithymia, there were no significant differences.

### Adjusted logistic regression analysis

In the bivariable analysis, weight categories, female sex, mothers’ education level, depression, anxiety, VGA, and FBA showed a significant association with HBP (Supplementary Table 3). Regarding the multivariate analysis, BMI categories were significant independent risk factors for HBP, with obesity and overweight as the most relevant risk factors [OR=3.73 (2.55-5.41) for obesity and OR = 1.72 (1.18–2.51) for overweight; *p* value < 0.001 and 0.008, respectively; Table 3].

**Table 3:** Factors associated with hypertension in secondary school adolescents in Sousse, Tunisia, 2017–2018.

	Total Population (*n* = 1392)			
	OR	95% CI	*p* value	
Age (years)	0.892	0.785	1.015	**0.078**
Female sex	0.410	0.298	0.563	**<0.001**
Depression	0.678	0.518	0.886	**0.008**
Weight status				
Overweight	1.723	1.183	2.511	**0.008**
Obesity	3.738	2.558	5.416	**<0.001**

Female sex [OR= 0.41 (0.29–0.56), *p* value < 0.001] and depression [OR = 0.67 (0.51–0.88), *p* = 0.008] were independent protective factors for HBP.

There were no differences in BMI between depressed and nondepressed students or collinearity. Mothers’ education level, anxiety, VGA, and FBA were no longer associated in the adjusted multivariate analysis. The results of the fully adjusted logistic regression model are shown in Table 2.

## Discussion

In our screening study of Tunisian adolescents, we found a high prevalence of HBP of approximately 15.4%, with a higher prevalence in boys than in girls. We also found that the most significant contributors to HBP were obesity and overweight, which could be related to certain lifestyle habits. In addition, we noted that certain psychological or sociodemographic aspects might play a role in the development of HBP.

Our high prevalence of HBP is in line with the prevalence found in some previous studies with similar characteristics in developing countries. Studies performed in India [10] and Turkey [11]showed hypertension prevalences of 21.5% and 14.8%, respectively. In contrast, studies conducted among young individuals in some developed countries showed significantly lower hypertension prevalences; for instance, the hypertension prevalence was 2.53% in Hungary [12] and approximately 7–10% in Canada [13].

Comparing prevalence rates between countries can be problematic for several reasons, such as differences in BP measurement protocols, differences in baseline characteristics of the study population, and the use of different criteria for hypertension definition [11]. For example, in some of the cited studies with higher hypertension prevalences, the 2017 AAP clinical paediatric guideline was used to define hypertension, which is known to be responsible for increased hypertension prevalences [14].

Furthermore,it was shown that the prevalence of hypertension may be overestimated if BP is measured only on a single occasion, such as in our study. In the literature, repeated BP measurements were followed by a decrease in the prevalence of hypertension [15]. For instance, a study conducted among American schoolchildren showed at the first assessment, the prevalence of hypertension was 19.4%. After 1–2 weeks, only 9.5% of the schoolchildren were considered to have hypertension, and in a third evaluation, the prevalence dropped to 4.5% [16]. This finding was consistent with another study conducted in Iceland including 970 students aged 9 to 10 years. At the initial screening, 13.1% of students had hypertension, whereas the prevalence was 6% after the second screening and only 3.1% following the third screening [17]. This finding implies that the reported hypertension prevalence in our study may decrease with repeated BP measurements at more than one visit. These facts highlight the importance of the surveillance of BP from an early age and the role of longitudinal studies in the estimation of the prevalence of sustained true hypertension.

As reported in the literature, our prevalence of HBP was also significantly higher in boys than in girls, reaching a prevalence of approximately 22.9% in the former group. Experimental studies showed that androgen hormones might play an essential role in BP differences between males and females. There are several possible mechanisms by which androgens may increase BP, mainly through increased activation of the renin-angiotensin system [18].

The prevalence of HBP was significantly higher in students younger than 16 years than in those who were older. This finding could be due to selection bias or the significantly lower prevalence of some factors associated with hypertension, such as depression. Furthermore, the potential impact of BP classification based on percentile in this group may have played a role.

A large proportion of students with hypertension had ISH. This finding could be related to the amplification phenomenon, which is not uncommon in youth and refers to those with normal central SBP but elevated brachial SBP, so-called “spurious hypertension.” Unfortunately, we did not have the equipment needed to measure the central BP to rule out this possibility. The clinical significance of this category is currently unknown, and further studies are needed in this field [19].

Our study reported a positive association between overweight and obesity and hypertension. The relationship between obesity and hypertension is well established. Several studies recognized this association in children and adults [20, 21]. Furthermore, the effects of weight reduction on BP are well documented in the literature [22]. The parallel increase in BP levels with increasing BMI may be related to some adipocyte-derived factors. These released factors have been linked to BP control. Aberrant production and release of these factors may be responsible for the high hypertension prevalence in obese individuals [23]. Among the proposed mechanisms unique to obesity-associated hypertension, we mention renal medulla changes, genetics, and metabolic factors [24].

Following a healthy lifestyle, regular PA and the maintenance of a healthy diet throughout life are crucial to preventing cardiovascular diseases (CVDs). However, developing countries are far from following the recommended healthy diet needed to prevent CVD. In Mediterranean countries, which are traditionally known for their healthy traditional dishes, including a high consumption of vegetables, fibre, and fish, there is a trend to adopt unhealthier eating habits [25]. Therefore, the prevalence of overweight and obesity, even in children, is increasing, exposing individuals to the risk of developing hypertension [26]. In our study, none of the studied lifestyle factors, including time spent on the internet, PA, FVI, and fast-food consumption were independent risk factors for hypertension. The lack of an association could be related to the collinearity of some of the included variables and complex interactions among lifestyle factors [27].

Concerning addictive behaviours, other than VGA and FA, no association was found between HBP and smoking, drinking, or illicit substance use. Although HBP in the bivariable analysis was significantly higher in students with VGA, this association disappeared after adjusting for sex. VGA can be considered a risk factor for sedentarism. We did not have information about the average time per week for the comparison of VGA and FA. A German study demonstrated a significant increase in SBP and DBP while playing video games (VGs) [28]. These effects may become exacerbated and persistent when VGs are played frequently over time. To understand the mechanisms that may link VGA to hypertension, some studies have highlighted the positive association between VGA and obesity [29]. Other studies explained this association through increased consumption of energy-dense snack foods while playing VGs [30], leading to an increase in body weight.

Regarding mental factors, students with anxiety and depression showed a lower prevalence of hypertension. These findings somehow contradict the growing knowledge showing the association of many emotional and cognitive issues with hypertension [31]. Inverse effects of the same psychological factors on BP are also possible [32]. Few studies have examined the association of mental conditions with BP among youth. Conversely, some studies in elderly individuals have shown that depression was associated with low BP [33]. In other studies, the authors found low BP to be a risk factor for depression instead of a consequence [34]. The relationship between depression and hypertension is biologically plausible and could be explained through autonomic nervous system dysfunction, which occurs in both depression [35] and hypertension [36].

### Strengths and limitations of the study

One of the main limitations of our study is its cross-sectional design, which does not allow us to determine the time sequence between the development of hypertension and its associated factors or to establish a causal relationship. Also, as the participants are evaluated at a single point, misclassification and selection bias are possible. Moreover, some of the associations are difficult to explain in cross-sectional studies. As for the quasi-experimental design without a control group, the health impact of our screening intervention cannot be assessed. However, this type of study, especially in children, could outline the problem and guide policy-makers in implementing screening and prevention programmes. To determine the temporal sequence between hypertension and its associated factors, it is necessary to carry out prospective studies because some comorbidities would not be diagnosed until later in adulthood. The causal mechanisms are not yet fully understood, but they are likely to involve a complex interplay of biological, psychological, and social factors.

Another major limitation was that due to the screening purpose of the study and logistics, only one BP reading was performed, which could lead to misclassification. Measurements on 2 or more occasions are operationally hard to perform in population studies. However, most students had BP values within the normal range, and unusually, the second and third BP measurements were higher than the first. Moreover, BP was repeated for those within the high-normal or hypertensive range, and the lowest measurement was selected. Although it has not been explicitly validated in children, the oscillometric BP device used in our study has been validated in adults, and as most included students were close to adult age, we believe the results are reliable. Additionally, the normal distribution of BP in the sample supports the accuracy of BP measurements. Our protocol served the screening purpose of the study, and it had a very high sensitivity, but at the cost of false-positives. False-positives would include those who would have had normal blood pressure if we had used the standard protocol of three readings, white coat hypertension, and the previously mentioned spurious hypertension. White coat hypertension is not without risk and has been associated with a higher risk of CVD and total mortality [37]. The prevalence of sustained hypertension, confirmed with BP measurements in three office visits, is expected to be lower than that found in our population study [1]. Screening programmes help in the examination of population trends, and most epidemiological studies have used a single BP reading for screening to calculate the hypertension prevalence [1]. However, further studies are needed to examine the prevalence of sustained, ‘true’ hypertension, confirmed with three BP readings.

The potential impact due to overestimation of hypertension has not been evaluated but the philosophy under a screening campaign is to increase the concern about a healthy problem, and early detection of cases, even at the cost of a high percentage of false positive. Studies of this kind are essential from a public health perspective because they can help underline the main health issues and risk behaviours associated with the development of cardiovascular risk factors and associated target organ damage. From a clinical perspective, improving health education during childhood and adolescence at a population level is probably the most cost-effective intervention to reduce the morbidity and the economic burden related to cardiovascular diseases.

Finally, we selected a representative sample of secondary school adolescents in Sousse, our results may be extrapolated to other regions of Tunisia or other similar Mediterranean LMIC, however, this fact remains to be proved. Additionally, the adolescent population in the age range of 10-14 y was not represented. Thus, our results are only well suited for older adolescents. Even when Sousse schools and classes were randomly selected, we cannot discard the possibility of some selection bias based on individual characteristics. As our students were not compared with those of the other non-selected classes or schools, we cannot identify those factors related to potential selection bias.

Among the main strengths of our research is that we used a survey design to obtain the most representative sample of adolescents for the region, and our sample size was large enough to obtain a reliable estimate of the prevalence of HBP. Although we decided to exclude students with unexpectedly very HBP levels to rule out the possibility of measurement bias or secondary forms of hypertension, our main results remained invariable when including those subjects.

Our survey is one of the most extensive performed in Tunisia and includes a wide range of relevant information, including sociodemographic data, mental conditions, and addictions. Our study is unique in the sense that has analysed the interaction of BP with several sociodemographic, mental conditions, and addictions in a transition stage between childhood and adulthood. A detailed picture of Sousse adolescents has been collected and can aid in the identification of health problems suitable for the development of preventive programmes and the allocation of resources by Tunisian health authorities.

As the real impact of the screening campaigns in children and adolescents on health is unknown, further research on the impact of these interventions with longitudinal follow-up is urgently needed. More resources should be allocated to ease the primordial prevention in the cardiovascular field.

## Conclusions

Elevated BP within the hypertension range was found to have a high prevalence among adolescents and to be strongly associated with some modifiable factors, such as overweight and obesity. The morbidity and mortality attributable to hypertension are largely preventable through prevention, education, and the control of its risk factors. These actions are more likely to be effective if implemented at the population level within a multisectoral approach involving all social, political, and economic actors. The targeting of children and adolescents at school through education about the impact of lifestyles on health is probably the most cost-effective way to reduce the burden of CVD in the long term.

### Summary table

#### What is known about topic

- The prevalence of hypertension is rising among young subjects and evidence suggests that it tracks from childhood
- The morbidity and mortality attributable to hypertension are largely preventable through prevention, education, and the control of its risk factors.
- LMICs are experiencing demographic and epidemiological transition with a groing burden of chronic conditions.

#### What this study adds

- Elevated blood pressure within the hypertension range was found to have a high prevalence among adolescents and to be strongly associated with some modifiable factors, such as overweight and obesity.
- There is an urgent need to develop multisectoral approach to prevent CVD risk factors in low income settings at an early age.
- Targeting children and adolescents at school through education about the impact of lifestyles on health may bea cost-effective way to reduce the burden of CVD in the long-term.

### Supplementary information

Supplemental material

## Data Availability

The data used to support the findings of this study are available from the corresponding author upon request.
