# Supplementary material for: The prevalence of high blood pressure and its determinants among Tunisian adolescents
Source: J Hum Hypertens. 2022 Apr 8;38(4):371–9. doi: 10.1038/s41371-022-00677-x (PMC11001578; doi:10.1038/s41371-022-00677-x)
Supplement: Supplementary file 1 — Supplemental material [file 41371_2022_677_MOESM1_ESM.docx]

**Secondary school**:......................................... **Survey date**: 2018 (__)(__)

**Questionnaire in secondary schools**

Greetings and peace: This questionnaire is designed for a study on the risk factors for chronic diseases and mental health in the school environment conducted by the Department of Epidemiological Sciences at Farhat Hached Hospital in Sousse.

Therefore, we ask you to fill it, assuring you that your answers will be treated with absolute confidentiality. It is requested that you do not write your name on this questionnaire.

Thank you.

**1-** Date of birth: (___).(___). (______)

**2**- Sex 🞎 Boy 🞎 Girl

**3**- Class :..........................................

**4-** Ordinal number: (____)

**5-** Section: (for the second, third and fourth year)

| For the second year | For the third and fourth year | |
| --- | --- | --- |
| 🞎 Science  🞎 Literature  🞎 Computer science 🞎 Sports | 🞎 Natural science  🞎 Maths  🞎 Literature  🞎 Technical science | 🞎 Economy and management  🞎 Sports  🞎 Computer science |

**6- Have you repeated a school year** Yes 🞎 No 🞎

**If yes, cite the number of years**: 🞎 **secondary**.....year(s) 🞎**preparatory**......year(s) 🞎**primary**....... year(s)

**7- The Rosenberg Self-Esteem Scale:** Below is a list of statements dealing with your general feelings about yourself. There are four possible answers for each of these 10 questions, from "strongly agree" to "strongly disagree. Tap the box to indicate how strongly you agree or disagree with each statement.

|  | **Strongly Agree** | **Agree** | **Disagree** | **Strongly Disagree** |
| --- | --- | --- | --- | --- |
| 1. On the whole, I am satisfied with myself. | 🞎 | 🞎 | 🞎 | 🞎 |
| 1. At times I think I am no good at all. | 🞎 | 🞎 | 🞎 | 🞎 |
| 1. I feel that I have a number of good qualities. | 🞎 | 🞎 | 🞎 | 🞎 |
| 1. I am able to do things as well as most other people. | 🞎 | 🞎 | 🞎 | 🞎 |
| 1. I feel I do not have much to be proud of. | 🞎 | 🞎 | 🞎 | 🞎 |
| 1. I certainly feel useless at times. | 🞎 | 🞎 | 🞎 | 🞎 |
| 1. I feel that I'm a person of worth, at least on an equal plane with others | 🞎 | 🞎 | 🞎 | 🞎 |
| 1. I wish I could have more respect for myself | 🞎 | 🞎 | 🞎 | 🞎 |
| 1. All in all, I am inclined to feel that I am a failure | 🞎 | 🞎 | 🞎 | 🞎 |
| 1. I take a positive attitude toward myself | 🞎 | 🞎 | 🞎 | 🞎 |

**8- Beck's Depression Inventory:** Read every sentence and choose the one that best describes your mental state during the last seven days including today. Mark only one answer that describes your feelings.

| **A** |  | 0 | I do not feel sad. |
| --- | --- | --- | --- |
|  |  | 1 | I feel sad |
|  |  | 2 | I am sad all the time and I can't snap out of it. |
|  |  | 3 | I am so sad and unhappy that I can't stand it. |
| **B** |  | 0 | I am not particularly discouraged about the future. |
|  |  | 1 | I feel discouraged about the future. |
|  |  | 2 | I feel I have nothing to look forward to. |
|  |  | 3 | I feel the future is hopeless and that things cannot improve. |
| **C** |  | 0 | I do not feel like a failure. |
|  |  | 1 | I feel I have failed more than the average person. |
|  |  | 2 | As I look back on my life, all I can see is a lot of failures. |
|  |  | 3 | I feel I am a complete failure as a person. |
| **D** |  | 0 | I get as much satisfaction out of things as I used to. |
|  |  | 1 | I don't enjoy things the way I used to. |
|  |  | 2 | I don't get real satisfaction out of anything anymore. |
|  |  | 3 | I am dissatisfied or bored with everything. |
| **E** |  | 0 | I don't feel particularly guilty |
|  |  | 1 | I feel guilty a good part of the time. |
|  |  | 2 | I feel quite guilty most of the time. |
|  |  | 3 | I feel guilty all of the time. |
| **F** |  | 0 | I don't feel I am being punished. |
|  |  | 1 | I feel I may be punished. |
|  |  | 2 | I expect to be punished. |
|  |  | 3 | I feel I am being punished. |
| **G** |  | 0 | I don't feel disappointed in myself. |
|  |  | 1 | I am disappointed in myself. |
|  |  | 2 | I am disgusted with myself. |
|  |  | 3 | I hate myself. |
| **H** |  | 0 | I don't feel I am any worse than anybody else. |
|  |  | 1 | I am critical of myself for my weaknesses or mistakes. |
|  |  | 2 | I blame myself all the time for my faults. |
|  |  | 3 | I blame myself for everything bad that happens. |
| **I** |  | 0 | I don't have any thoughts of killing myself. |
|  |  | 1 | I have thoughts of killing myself, but I would not carry them out. |
|  |  | 2 | I would like to kill myself. |
|  |  | 3 | I would kill myself if I had the chance. |
| **J** |  | 0 | I don't cry any more than usual. |
|  |  | 1 | I cry more now than I used to. |
|  |  | 2 | I cry all the time now. |
|  |  | 3 | I used to be able to cry, but now I can't cry even though I want to. |
| **K** |  | 0 | I am no more irritated by things than I ever was. |
|  |  | 1 | I am slightly more irritated now than usual. |
|  |  | 2 | I am quite annoyed or irritated a good deal of the time. |
|  |  | 3 | I feel irritated all the time. |
| **L** |  | 0 | I have not lost interest in other people. |
|  |  | 1 | I am less interested in other people than I used to be. |
|  |  | 2 | I have lost most of my interest in other people. |
|  |  | 3 | I have lost all of my interest in other people. |
| **M** |  | 0 | I make decisions about as well as I ever could. |
|  |  | 1 | I put off making decisions more than I used to. |
|  |  | 2 | I have greater difficulty in making decisions more than I used to. |
|  |  | 3 | I can't make decisions at all anymore. |

**9-** **Game Addiction Scale**: Using the grid below, mark with (X) the appropriate box. Do not put more than an (X) mark on the same statement (Video games: all the games you play, whether on the phone or on the computer)

| **During the last 6 months** | **very often** | **often** | **sometimes** | **Rarely** | **never** |
| --- | --- | --- | --- | --- | --- |
| 1. Did you think about playing a game all day long? | 🞎 | 🞎 | 🞎 | 🞎 | 🞎 |
| 1. Did you spend much free time on games? | 🞎 | 🞎 | 🞎 | 🞎 | 🞎 |
| 1. Have you felt addicted to a game? | 🞎 | 🞎 | 🞎 | 🞎 | 🞎 |
| 1. Did you play longer than intended? | 🞎 | 🞎 | 🞎 | 🞎 | 🞎 |
| 1. Did you spend increasing amounts of time on games? | 🞎 | 🞎 | 🞎 | 🞎 | 🞎 |
| 1. Were you unable to stop once you started playing? | 🞎 | 🞎 | 🞎 | 🞎 | 🞎 |
| 1. Did you play games to forget about real life?* | 🞎 | 🞎 | 🞎 | 🞎 | 🞎 |
| 1. Have you played games to release stress? | 🞎 | 🞎 | 🞎 | 🞎 | 🞎 |
| 1. Have you played games to feel better? | 🞎 | 🞎 | 🞎 | 🞎 | 🞎 |
| 1. Were you unable to reduce your game time? | 🞎 | 🞎 | 🞎 | 🞎 | 🞎 |
| 1. Have others unsuccessfully tried to reduce your game use?* | 🞎 | 🞎 | 🞎 | 🞎 | 🞎 |
| 1. Have you failed when trying to reduce game time? | 🞎 | 🞎 | 🞎 | 🞎 | 🞎 |
| 1. Have you felt bad when you were unable to play?* | 🞎 | 🞎 | 🞎 | 🞎 | 🞎 |
| 1. Have you become angry when unable to play? | 🞎 | 🞎 | 🞎 | 🞎 | 🞎 |
| 1. Have you become stressed when unable to play? | 🞎 | 🞎 | 🞎 | 🞎 | 🞎 |
| 1. Did you have fights with others (e.g., family, friends) over your time spent on games?* | 🞎 | 🞎 | 🞎 | 🞎 | 🞎 |
| 1. Have you neglected others (e.g., family, friends) because you were playing games? | 🞎 | 🞎 | 🞎 | 🞎 | 🞎 |
| 1. Have you lied about time spent on games? | 🞎 | 🞎 | 🞎 | 🞎 | 🞎 |
| 1. Has your time on games caused sleep deprivation? | 🞎 | 🞎 | 🞎 | 🞎 | 🞎 |
| 1. Have you neglected other important activities (e.g., school, work, sports) to play games?* | 🞎 | 🞎 | 🞎 | 🞎 | 🞎 |
| 1. Did you feel bad after playing for a long time? | 🞎 | 🞎 | 🞎 | 🞎 | 🞎 |

1. **The 20-Item Toronto Alexithymia Scale III.** Indicate, using the grid below, the response that applies to you. Mark with (X) the appropriate box. Do not put more than an (X) mark on the same statement.

|  | **strongly agree** | **Moderately agree** | **Neither disagree nor agree** | **Moderately disagree** | **strongly disagree** |
| --- | --- | --- | --- | --- | --- |
| 1. I am often confused about what emotion I am feeling | 🞎 | 🞎 | 🞎 | 🞎 | 🞎 |
| 1. It is difficult for me to find the right words for my feelings | 🞎 | 🞎 | 🞎 | 🞎 | 🞎 |
| 1. I have physical sensations that even doctors don’t understand | 🞎 | 🞎 | 🞎 | 🞎 | 🞎 |
| 1. I am able to describe my feelings easily | 🞎 | 🞎 | 🞎 | 🞎 | 🞎 |
| 1. I prefer to analyse problems rather than just describe them | 🞎 | 🞎 | 🞎 | 🞎 | 🞎 |
| 1. When I am upset, I don’t know if I am sad, frightened, or angry | 🞎 | 🞎 | 🞎 | 🞎 | 🞎 |
| 1. I am often puzzled by sensations in my body | 🞎 | 🞎 | 🞎 | 🞎 | 🞎 |
| 1. I prefer just to let things happen rather than to understand why they turned out that way | 🞎 | 🞎 | 🞎 | 🞎 | 🞎 |
| 1. I have feelings that I can’t quite identify | 🞎 | 🞎 | 🞎 | 🞎 | 🞎 |
| 1. Being in touch with emotions is essential | 🞎 | 🞎 | 🞎 | 🞎 | 🞎 |
| 1. I find it hard to describe how I feel about people | 🞎 | 🞎 | 🞎 | 🞎 | 🞎 |
| 1. People tell me to describe my feelings more | 🞎 | 🞎 | 🞎 | 🞎 | 🞎 |
| 1. I don’t know what’s going on inside me | 🞎 | 🞎 | 🞎 | 🞎 | 🞎 |
| 1. I often don’t know why I am angry | 🞎 | 🞎 | 🞎 | 🞎 | 🞎 |
| 1. I prefer talking to people about their daily activities rather than their feelings | 🞎 | 🞎 | 🞎 | 🞎 | 🞎 |
| 1. I prefer to watch “light” entertainment shows rather than psychological dramas | 🞎 | 🞎 | 🞎 | 🞎 | 🞎 |
| 1. It is difficult for me to reveal my innermost feelings, even to close friends | 🞎 | 🞎 | 🞎 | 🞎 | 🞎 |
| 1. I can feel close to someone, even in moments of silence | 🞎 | 🞎 | 🞎 | 🞎 | 🞎 |
| 1. I find examination of my feelings useful in solving personal problems | 🞎 | 🞎 | 🞎 | 🞎 | 🞎 |
| 1. Looking for hidden meanings in movies or plays distracts from their enjoyment | 🞎 | 🞎 | 🞎 | 🞎 | 🞎 |

1. **The Screen for Child Anxiety Related Emotional Disorders (SCARED):** Below is a list of statements about yourself. Mark with (X) the response that applies to your opinion. Answer each statement honestly; there is no right or wrong answer.

| **Very True** | **Somewhat True** | **Not True** |  |
| --- | --- | --- | --- |
| 🞎 | 🞎 | 🞎 | 1- When I feel frightened, it is hard to breathe |
| 🞎 | 🞎 | 🞎 | 2- I get headaches when I am at school |
| 🞎 | 🞎 | 🞎 | 3- I don’t like to be with people I don’t know well |
| 🞎 | 🞎 | 🞎 | 4- I get scared if I sleep away from home. |
| 🞎 | 🞎 | 🞎 | 5- I worry about other people liking me |
| 🞎 | 🞎 | 🞎 | 6- When I get frightened, I feel like passing out |
| 🞎 | 🞎 | 🞎 | 7. I am nervous |
| 🞎 | 🞎 | 🞎 | 8. I follow my mother or father wherever they go |
| 🞎 | 🞎 | 🞎 | 9- People tell me that I look nervous. |
| 🞎 | 🞎 | 🞎 | 10- I feel nervous with people I don’t know well |
| 🞎 | 🞎 | 🞎 | 11- I get stomach-aches at school. |
| 🞎 | 🞎 | 🞎 | 12- When I get frightened, I feel like I am going crazy |
| 🞎 | 🞎 | 🞎 | 13- I worry about sleeping alone |
| 🞎 | 🞎 | 🞎 | 14- I worry about being as good as other kids |
| 🞎 | 🞎 | 🞎 | 15- When I get frightened, I feel like things are not real |
| 🞎 | 🞎 | 🞎 | 16- I have nightmares about something bad happening to my parents |
| 🞎 | 🞎 | 🞎 | 17- I worry about going to school. |
| 🞎 | 🞎 | 🞎 | 18- When I get frightened, my heart beats fast |
| 🞎 | 🞎 | 🞎 | 19- I get shaky |
| 🞎 | 🞎 | 🞎 | 20- I have nightmares about something bad happening to me |
| 🞎 | 🞎 | 🞎 | 21- I worry about things working out for me |
| 🞎 | 🞎 | 🞎 | 22- When I get frightened, I sweat a lot |
| 🞎 | 🞎 | 🞎 | 23- I am a worrier |
| 🞎 | 🞎 | 🞎 | 24- I get really frightened for no reason at all |
| 🞎 | 🞎 | 🞎 | 25- I am afraid to be alone in the house |
| 🞎 | 🞎 | 🞎 | 26- It is hard for me to talk with people I don’t know well |
| 🞎 | 🞎 | 🞎 | 27- When I get frightened, I feel like I am choking |
| 🞎 | 🞎 | 🞎 | 28- People tell me that I worry too much |
| 🞎 | 🞎 | 🞎 | 29- I don’t like to be away from my family |
| 🞎 | 🞎 | 🞎 | 30- I am afraid of having anxiety (or panic) attacks |
| 🞎 | 🞎 | 🞎 | 31- I worry that something bad might happen to my parents |
| 🞎 | 🞎 | 🞎 | 32- I feel shy with people I don’t know well |
| 🞎 | 🞎 | 🞎 | 33- I worry about what is going to happen in the future |
| 🞎 | 🞎 | 🞎 | 34- When I get frightened, I feel like throwing up |
| 🞎 | 🞎 | 🞎 | 35- I worry about how well I do things |
| 🞎 | 🞎 | 🞎 | 36- I am scared to go to school |
| 🞎 | 🞎 | 🞎 | 37- I worry about things that have already happened |
| 🞎 | 🞎 | 🞎 | 38- When I get frightened, I feel dizzy |
| 🞎 | 🞎 | 🞎 | 39- I feel nervous when I am with other children or adults and I have to do something while they watch me (for example: read aloud, speak, play a game, play a sport). |
| 🞎 | 🞎 | 🞎 | 40- I feel nervous when I am going to parties, dances, or any place where there will be people that I don’t know well |
| 🞎 | 🞎 | 🞎 | 41- I am shy |

**12- Facebook Addiction Scale:** Using the grid below, mark with (X) the response that applies to you. Do not put more than an (X) mark on the same statement.

|  | **Very rarely** | **Rarely** | **Sometimes** | **Often** | **Very often** |
| --- | --- | --- | --- | --- | --- |
| 1. Spent a lot of time thinking about Facebook or planned use of Facebook | 🞎 | 🞎 | 🞎 | 🞎 | 🞎 |
| 1. Felt an urge to use Facebook more and more | 🞎 | 🞎 | 🞎 | 🞎 | 🞎 |
| 1. Used Facebook in order to forget about personal problems | 🞎 | 🞎 | 🞎 | 🞎 | 🞎 |
| 1. Tried to cut down on the use of Facebook without success | 🞎 | 🞎 | 🞎 | 🞎 | 🞎 |
| 1. Become restless or troubled if you have been prohibited from using Facebook | 🞎 | 🞎 | 🞎 | 🞎 | 🞎 |
| 1. Used Facebook so much that it has had a negative impact on your job/studies | 🞎 | 🞎 | 🞎 | 🞎 | 🞎 |

**13- How much time do you spend surfing the internet per day? .....hours**..........**minutes**

**14- Do you have Facebook** 🞎 yes 🞎No

**If yes, what is the average time you spend on Facebook per day?**

.... **hours**.......... **minutes**

1. **What is your current weight?** .............. kg 🞎 Don't know
2. **What is your current height?**................ m 🞎 Don't know
3. **In your opinion, how do you estimate your weight?**

| 🞎underweight | 🞎 overweight | 🞎normal weight | 🞎 don’t know |
| --- | --- | --- | --- |

1. **What is the educational level of your mother?**

| 🞎 Illiterate | 🞎 Secondary education | 🞎 Don't know |
| --- | --- | --- |
| 🞎 Primary education | 🞎 Higher Education |  |

1. **What is the educational level of the father?**

| 🞎 Illiterate | 🞎 Secondary education | 🞎 Don't know |
| --- | --- | --- |
| 🞎 Primary education | 🞎 Higher Education |  |

1. **What is your mother's profession?**

| 🞎 Unemployed | 🞎 Employee |
| --- | --- |
| 🞎 Labourer | 🞎 Senior staff |
| 🞎 Private sector | 🞎 Other..................... |

1. **What is your father 's profession?**

| 🞎 Unemployed | 🞎 Employee |
| --- | --- |
| 🞎 Labourer | 🞎 Senior staff |
| 🞎 Private sector | 🞎 Other..................... |

1. **Do you eat 5 vegetables and fruits per day** 🞎 yes 🞎no
2. During the past week, how many days did you eat fast food

| 🞎 never | 🞎 2 days | 🞎 4 days | 🞎 6 days |
| --- | --- | --- | --- |
| 🞎one day | 🞎 3 days | 🞎 5 days | 🞎 days |

1. **What is your average weekly pocket money?** .............. Dinars
2. **Physical activity: In your opinion, do you practice at least 60 minutes of physical activity per day for 5 or more days per week? (**Any activity that increases your heart rate and makes you breathless for some time. Physical activity can be sports, playing with friends, jogging, running, dancing and playing soccer**.** 🞎 Yes 🞎No
3. **Have you ever smoked a cigarette or tried to smoke at least once in your life?** 🞎 yes 🞎 no
4. **What was your age when you first smoked a cigarette?.**.................. year(s) 🞎 I have never smoked
5. **During the last month, how many days did you smoke cigarettes?**

| 🞎 Ihave never smoked | | |
| --- | --- | --- |
| 🞎1–2 days | 🞎 6-9 days | 🞎 20-29 days |
| 🞎 3-5 days | 🞎 10-19 days | 🞎 30 days |

1. **During the days you smoked in the last month, how many cigarettes did you usually smoke?**

| 🞎 I didn’t smoke | 🞎 6-10 cigarettes a day | 🞎 11-20 cigarettes a day |
| --- | --- | --- |
| 🞎 less than one cigarette | 🞎 2-5 cigarettes a day | 🞎more than cigarettes a day |

1. **During the last month, how many days did you use water pipe?**

| \| 🞎 I have never smoked \| \| \| \| --- \| --- \| --- \| \| 🞎 1-2 days \| 🞎 6-9 days \| 🞎 20-29 days \| \| 🞎 3-5 days \| 🞎 10-19 days \| 🞎 30 days \| |
| --- | --- | --- | --- | --- | --- | --- | --- | --- | --- |

1. **During the last month, how many days did you use an e-cigarette or a vape?**

| 🞎 Ihave never smoked | | |
| --- | --- | --- |
| 🞎 1-2 days | 🞎 6-9 days | 🞎 29-20 days |
| 🞎 3-5 days | 🞎 19-10 days | 🞎 30 days |

1. **Do you consume alcoholic drinks (beer, beer, wine, whiskey, etc.)** 🞎 Yes 🞎No
2. **Usually, how many days do you drink alcohol? (**One drink is equivalent to a bottle or can of beer or a glass of wine or a shot of whiskey.)

| 🞎 every day | 🞎1-3 days a month |
| --- | --- |
| 🞎 5-6 days a week | 🞎 in special occasions |
| 🞎 1-4 days a week | 🞎 I don’t drink |

1. **During the days you drank alcohol, how many drinks did you average per day?** number............... 🞎I don’t drink
2. **How old were you when you first drank alcohol?** …................. years 🞎 I have never drunk
3. **Have you ever used an illicit substance even once in your life? (multiple choices)**

| 🞎No, I have never used illicit substance | 🞎 Ecstasy |
| --- | --- |
| 🞎Inhalants (solvents, glue, paint.) | 🞎 (Subutex |
| 🞎Cannabis | 🞎 (Madkok |
| 🞎 Pills (Artane) | 🞎 (LSD |
| 🞎 Cocaine/Crack | 🞎 Ketamine |
| 🞎 Heroin | 🞎 (Fentanyl |
| 🞎 Other substances, cite it............................................................... | |

1. **How old were you when you used drugs for the first time?** ….................old 🞎 I have never used drugs
2. **How many times have you used drugs in your life?**

| 🞎 Never |  | 🞎3 to 9 times |  | 🞎 More than 20 times |
| --- | --- | --- | --- | --- |
| 🞎Once or twice |  | 🞎10 to 19 times |  | 🞎 Other answer................. |

**- end of Survey -**

**Thank you for answering**

**For the medical team:**

| **mmHg** | **…………………** | **SBP** | **kg** | **…………………** | **Weight** |
| --- | --- | --- | --- | --- | --- |
| **mmHg** | **…………………** | **DBP** | **M** | **…………………** | **Height** |

Variable DefinitionS:

**Videogame addiction (VGA):** We used a 21-item questionnaire representing seven DSM-based criteria for game addiction: salience, tolerance, mood modification, withdrawal, relapse, conflict and problems. This questionnaire evaluates behaviour over the last six months, and responses are rated on a 5-point Likert scale (1=never, 2=very rarely, 3=sometimes, 4=often, 5=very often). The overall score ranges from 21 to 105. Higher scores reflect higher levels of VGA. Adolescents who scored 3 points or more on at least 4 of the 6 items were considered to have a VGA.

**Facebook addiction:** To assess Facebook addiction disorder, we used a brief version of the BFAS, screening behaviour over the last year. It includes six items reflecting the basic elements of addiction. Responses are rated on a 5-point Likert scale (1=very rarely, 2=rarely, 3=sometimes, 4=often, 5=very often)**.** The total score can range from six to thirty. Students who scored 3 points or more on at least 4 of the 6 items were considered to have a Facebook addiction.

**Self-esteem:** We used the RSE to measure self-esteem. Each item in this questionnaire has four response options (1=strongly agree, 2=agree, 3=disagree, 4=strongly disagree), offering a scale score range from 10 to 40.

**Depression:** To measure depression, we used the Arabic version of the BDI-II scale, which contains 42 items rated on a 4-point Likert scale (0=did not apply to me at all, 3=applied to me very much or most of the time). The scale score ranges from zero to 39, and higher scores indicate a higher level of depression.

**Emotional self-awareness (Alexithymia):** The TAS-20 is the most commonly used instrument for assessing alexithymia, a term used to describe emotional self-awareness. The scale includes 21 items, and all items are rated on a 5-point Likert scale (1=strongly agree, 2=agree, 3=neither agree nor disagree, 4=disagree, 5=strongly disagree). A score between 52 and 60 indicates possible alexithymia, and a score ≥ 61 indicates alexithymia.

**Anxiety:** The SCARED-C was used to assess different types of anxiety disorders among adolescents. Each item is rated on a 3-point Likert-type scale as follows: 0 = (not true), 1 = (sometimes true) and 2 = (very true or often true). The total score was calculated by summing the responses of the 41 items. The total score ranges from 0 to 82.

A total score ranging between 25 and 30 indicates the possibility of anxiety disorder, and a total score of at least 31 indicates an anxiety disorder.
